# Cost-effectiveness assessment of liquid biopsy for early detection of lung cancer in Brazil

**DOI:** 10.1371/journal.pone.0328631

**Published:** 2025-07-29

**Authors:** Kátia Marie Senna, Ivan Ricardo Zimmermann, Márcia Gisele Santos da Costa, Bernardo Rangel Tura, José Ronyeryson dos Santos Evangelista, Marisa Santos

**Affiliations:** Centre of Technology Assessment in Health, National Institute of Cardiology, Rio de Janeiro, Brazil; University of Oxford Nuffield Department of Population Health, UNITED KINGDOM OF GREAT BRITAIN AND NORTHERN IRELAND

## Abstract

**Introduction:**

Lung cancer has a low survival rate due to late diagnosis, with most cases detected at advanced stages. Liquid biopsy, a non-invasive alternative to tissue biopsy, has emerged as a potential screening tool for early lung cancer detection. This study evaluates the cost-effectiveness of liquid biopsy screening using an autoantibody test (EarlyCDT-Lung®) in high-risk populations from the perspective of the Brazilian Unified Health System (SUS).

**Methods:**

A decision-analytic model was developed, combining a decision tree and a Markov model to compare two strategies: standard clinical diagnosis without screening and liquid biopsy screening followed by confirmatory diagnostics. Model inputs included test accuracy, treatment costs, and survival data derived from systematic reviews, national registries, and published economic evaluations. The primary outcome was the incremental cost-effectiveness ratio (ICER) per quality-adjusted life year (QALY). Sensitivity analyses assessed model robustness.

**Results:**

Liquid biopsy screening analysis of a hypothetical cohort of 1,000 individuals at high-risk of lung cancer resulted in an incremental cost of $ 570,120 and an incremental effectiveness of 7.56 QALYs, with an ICER of $ 75,435.63 per QALY gained. This result has far exceeded the willingness-to-pay threshold in Brazil ($ 7,017.54–21,052.62/QALY). Therefore, in case of assuming no significant cost reductions or accuracy improvements, the strategy would only become cost-effective in contexts where lung cancer prevalence exceeds 4.0%. Probabilistic sensitivity analysis confirmed a low likelihood of cost-effectiveness, even with varying test sensitivity, lung cancer prevalence, and cost assumptions.

**Discussion:**

The adoption of an autoantibody test as a liquid biopsy for early lung cancer screening in high-risk populations in Brazil led to increased costs and was not considered cost-effective under the thresholds commonly applied in the Brazilian context.

## Introduction

Lung cancer is a major global public health concern, with a low overall survival rate of approximately 18%, possibly linked to the absence of symptoms in the early stages and, consequently, late disease detection. In Brazil, the estimated incidence of lung cancer for the 2020–2022 period (2020 – INCA) is 30,200 new cases, with 17,760 men and 12,440 women, corresponding to an estimated risk of 16.99/100,000 men and 11.56/100,000 women [1,2]. Most cases are detected in late stages during the diagnostic investigation of suspicious symptoms or as an incidental finding in radiological chest imaging, given its resemblance to other less lethal diseases [3]. However, cases detected at an early stage can have a five-years survival rate reach up to 60% [4,5].

Since the 1960s, screening as a strategy to aid in the early diagnosis of lung cancer has been studied with a focus on identifying an ideal screening test that is both safe for patients and cost-effective for healthcare systems. Studies using chest X-rays combined with sputum cytology, followed by CT scans, have not shown significant results in reducing mortality from the disease [6,7]. In 2011, a multicenter Trial [8] showed a 20% reduction in lung cancer mortality in a high-risk population screened with low-dose computed tomography (LDCT).

Over the past decade, liquid biopsy has emerged as a minimally invasive adjunct for the early detection of tumors in individuals with suspected lung cancer. This technique analyzes small fragments of circulating tumor DNA (ctDNA) and microRNAs (miRNAs) present in bodily fluids, most commonly plasma, but also urine, saliva, and stool, to provides a real-time snapshot of the tumor’s genomic landscape, facilitating early diagnosis, patient stratification, and therapeutic decision-making [9]. Some studies have shown that liquid biopsy can be considered a useful test for the early detection of lung cancer [10–12].

In Brazil, according to the current Diagnostic and Therapeutic Guidelines for Lung Cancer, published in 2014 [13], there is no recommendation for lung cancer screening in asymptomatic individuals. Therefore, as a liquid biopsy may offer a promising, less invasive approach for early tumor detection, enhancing the precision of screening protocols and potentially improving patient outcomes. A cost-effectiveness analysis of a screening strategy for the early detection of lung cancer in high-risk individuals was conducted from the perspective of the Brazilian Unified Health System (SUS).

Recent publications have reinforced the growing interest in evaluating the cost-effectiveness of liquid biopsy in oncology. A systematic review by Fagery et al. highlighted the economic value of this approach across multiple cancer types, including lung cancer [14]. Additionally, Zhao et al. conducted a modeling study suggesting that the combination of a multi-cancer blood test with LDCT may represent a cost-effective strategy for lung cancer screening in high-risk populations [15]. These findings further support the relevance of exploring blood-based screening methods as scalable and potentially cost-effective alternatives within public health systems.

## Materials and methods

### Context

Considering the unavailability of validated and commercially available liquid biopsy tests in Brazil, an analysis was conducted based on a test already available in several other countries and recommended for early detection of lung cancer using LDCT. The selected test is an immunobiomarker panel composed of seven specific autoantibodies, utilizing the enzyme-linked immunosorbent assay (ELISA) method for lung cancer identification. In clinical settings, a CT may be used as a confirmatory test following a positive liquid biopsy.

To analyze the evidence, a systematic search was conducted in the electronic databases MEDLINE (via PubMed) and EMBASE, without restrictions on date or language, along with a search in the *ClinicalTrials.gov* database. The objective was to answer the following structured question: “Is a specific lung cancer autoantibody test an accurate and cost-effective method for early detection in high-risk individuals compared to no screening?”

To address this question, the primary objective of the literature search was to identify systematic reviews that evaluated the diagnostic accuracy of autoantibody tests for lung cancer, aiming to ensure a reliable and synthesized evidence base for model parameterization. The search process was structured based on the key elements recommended by the Preferred Reporting Items for Systematic Reviews and Meta-Analyses (PRISMA) guideline [16]. A completed PRISMA checklist has been provided as supplementary material (S1 Appendix).

**Fig 1:**
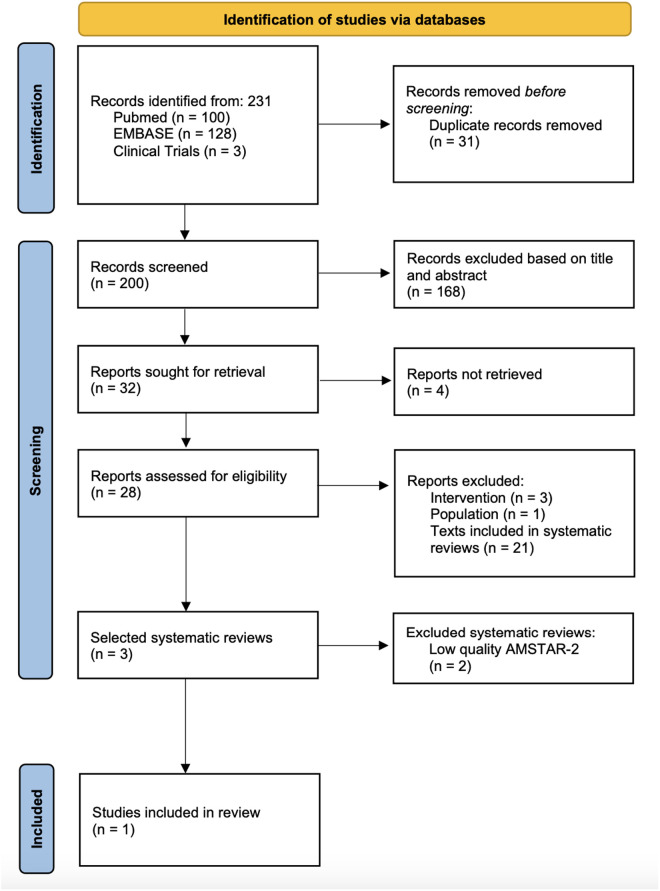
Systematic search and review process, conducted in accordance with the Preferred Reporting Items for Systematic Reviews and Meta-Analyses (PRISMA) Statement criteria.

Among the 228 studies retrieved, those in languages other than Portuguese, English, or Spanish were excluded. Three systematic reviews were selected for qualitative analysis (Fig 1). The selection and data extraction were carried out independently and blindly by two reviewers, with discrepancies resolved by consensus. The selection process was conducted using the free systematic review software *Rayyan QCRI*, available at: https://rayyan.qcri.org. Ultimately, three systematic reviews by Qin et al., 2018 [17], Tang, 2017 [18], and Chu, 2018 [19] were selected as they addressed the structured question. However, after evaluating methodological quality using the AMSTAR-2 tool [20], only Qin et al., 2018 [17] was included in the evidence analysis due to the low quality of the other two reviews.

None of the systematic reviews identified in our search addressed cost-effectiveness outcomes. Therefore, the economic evidence used in the model was obtained from separate studies specifically focused on economic evaluation, including the EarlyCDT-Lung® cost-effectiveness analysis by Edelsberg et al. (2018) [21]. Although the systematic review by Qin et al. (2018) [17] was included as the primary source of synthesized evidence, we used the Jett et al. (2014) [22] study to inform sensitivity and specificity estimates in the model because it reported real-world test performance of EarlyCDT-Lung® in a large clinical sample (n = 1,600), with disaggregated diagnostic parameters.

The systematic review by Qin et al., 2018 [17] aimed to assess the accuracy of autoantibody tests (AACs) for lung cancer diagnosis. The findings demonstrated that AACs have low sensitivity but high specificity as serum biomarkers for lung cancer. However, the data suggest that combining autoantibodies into panels may improve diagnostic performance. Based on their accuracy, the authors described these tests as most suitable for screening strategies to detect tumors in early stages [17]. Thus, an economic model was developed to evaluate the costs and consequences of using liquid biopsy with the EarlyCDT-Lung® autoantibody test as a screening strategy for early detection of lung cancer in high-risk populations. The following sections detail the steps and results of the economic evaluation model, in accordance with the Consolidated Health Economic Evaluation Reporting Standards (CHEERS) guideline [23].

Although LDCT has demonstrated clinical efficacy in reducing lung cancer mortality in high-risk populations, its implementation in Brazil remains limited by cost, availability, and lack of recommendation in national guidelines. At the time this analysis was conducted, no economic evaluations were available in the Brazilian context to inform decisions regarding LDCT adoption. Therefore, this study focused on assessing the potential value of introducing a blood-based liquid biopsy test as a screening strategy compared to no screening, reflecting the limited options available for early detection within the national healthcare system.

### Target population

The target population for the model was defined as all individuals at high risk for lung cancer, meeting the following criteria, established in cohort studies and validated in national and international guidelines: [3,24]

- Aged over 55 years
- Absence of significant respiratory symptoms
- Smoking history of more than 30 pack-years (current smokers or those who quit within the last 15 years)

For this model, NSCLC was used as the reference, given its representativeness of approximately 90% among different lung cancer types [25]. Screening eligibility was defined based on clinical risk factors, such as age and smoking history, without histological distinction at this stage. Although both NSCLC and SCLC may be present in the high-risk population, the model focused on NSCLC because it is the primary target of early detection strategies. Furthermore, the diagnostic performance of the EarlyCDT-Lung® test has been primarily validated for NSCLC.

### Study perspective

The study adopted the perspective of the Brazilian Unified Health System (SUS), considering all levels of funding (federal, state, and municipal). Reimbursement adjustments for procedures were applied according to the cost data section.

### Comparators

The diagnosis of lung cancer based on clinical suspicion, without prior screening, was considered the reference scenario. For Stage I and II cases, it was assumed that patients would undergo a LDCT scan and positron emission tomography-computed tomography (PET-CT). For suspected advanced-stage cases (Stage III and IV), bronchoscopy and percutaneous biopsy were also considered for proper disease staging [13].

The alternative strategy evaluated was the use of liquid biopsy with the EarlyCDT-Lung® test, in which individuals with a positive test result were assumed to proceed with confirmatory and staging procedures, although the specific diagnostic tests were not explicitly modeled. In clinical practice, such procedures typically include LDCT, PET-CT, bronchoscopy, and percutaneous biopsy, as recommended by national guidelines. The adoption of screening aims to detect cancer cases at an early stage, where prognosis and quality of life are significantly improved [2,26].

### Time horizon

The model adopts a lifetime horizon. For this, Brazilian life tables from 2017 were used, considering disease progression according to the average age of the cohort in the screening context and the natural history of the disease, which significantly impacts overall survival and quality of life [4].

### Discount rate

Following Brazilian methodological guidelines [27], an annual discount rate of 5% was applied to costs and outcomes in the base case, with variations from 0% to 10% in the sensitivity analysis.

### Outcome selection

The incremental cost-effectiveness ratio (ICER), which refers to the ratio of incremental differences of costs and outcomes between intervention and comparator, was calculated, considering absorbed costs in each arm and outcomes in quality-adjusted life years (QALY). The diagnostic events in the model (proportion of individuals diagnosed in Stage I or II) served as effectiveness determinants in terms of survival and quality of life (utilities).

### Effectiveness measurement

#### Accuracy.

The sensitivity and specificity data for the AAC, along with confidence intervals, were obtained from an accuracy study with more than 1,600 patients after a literature review [22]. The study estimated specificity: 87% (95% CI: 85–90%) and sensitivity: 41% (95% CI: 29–54%). The primary objective of the test was to increase early detection of individuals with Stage I and II cancer, a logic also adopted in the proposed model. As a result of early detection, 57% (n = 26/46) of patients were diagnosed at Stage I or II.

#### Utility.

For utility data in the cancer-free population, estimates were based on data available from a population-based study conducted in a Brazilian state (Federal District) [28]. Regarding early and advanced stages of lung cancer, due to the lack of Brazilian data, values were obtained from a systematic review and meta-analysis of lung cancer utilities, which included 23 international studies conducted in the United States, five in Canada, five in European countries, and two in Australia [26].

These utility values were assigned according to disease stage at diagnosis (early or advanced) and are assumed to reflect long-term average quality of life outcomes. They implicitly incorporate the effects of both disease progression and treatment burden by stage. This assumption has been adopted in previous economic models using stage-based utility differentiation.

### Resource and cost estimation

Costs were collected in Brazilian currency (R$) at 2024 values and converted to US dollars ($) at the exchange rate of R$5.70 per USD on February 14, 2025. Official sources were used to obtain SUS reimbursement values. The identification and quantification of cost items followed the NLST protocol [29] (USA, 2002–2010) and the Brazilian Diagnostic and Therapeutic Guidelines for Lung Cancer [13].

Although treatment effectiveness was not the study focus, costs related to treatment procedures were considered based on cancer staging.

The estimation of screening costs included expenses related to test acquisition (EarlyCDT-Lung®), sample collection, laboratory analysis, and follow-up medical consultations. These values were based on SUS reimbursement tables and institutional protocols from public hospitals, which already incorporate human resource costs related to clinical staff involved in outpatient procedures. For diagnostic confirmation after a positive screening result, we included imaging tests and procedures in accordance with national diagnostic and therapeutic guidelines for lung cancer, such as LDCT, PET-CT, bronchoscopy, and percutaneous biopsy. Costs were also based on official SUS reimbursement rates.

Treatment costs were stratified by disease stage. For advanced-stage cases (Stage III/IV), we used data from a detailed microcosting study conducted at a Brazilian public oncology reference center, which included costs for medical consultations, diagnostic exams, therapeutic procedures, hospitalizations, and pharmaceutical expenses, including analgesics and palliative care drugs. For early-stage cases (Stage I/II), costs were estimated using SUS reimbursement values applied to the standard treatment protocol, which includes surgical resection and possible adjuvant therapy. While less detailed than the microcosting study, these bundled reimbursement values implicitly include medical staff costs and basic pharmaceutical support. This approach reflects the structure of health service financing in the Brazilian public system.

#### Screening costs.

The direct costs were extracted from the SUS Management System Table of Procedures, Orthotics, Prosthetics, and Special Materials (SIGTAP) [30]. However, these values do not represent the actual procedure costs but rather the federal contribution portion.

Since SIGTAP values refer only to federal expense reimbursements, adjustments were applied based on average cost-sharing percentages from other government entities. These contributions range from 61% to 69% for procedures related to curative care in inpatient and hospital-day settings, respectively [31]. Therefore, a 2.84 adjustment factor was applied to the SIGTAP values used in the model.

The cost of the selected AAC test for analysis was obtained from Edelsberg et al., 2018 [21], a study funded by the manufacturer, which estimated the test price at $575.

#### Treatment costs.

After diagnosis, the average treatment costs in the hypothetical cohort were considered based on early-stage (Stage I or II) or advanced-stage disease.

#### Early-stage disease (stage I or II).

According to the Brazilian Diagnostic and Therapeutic Guidelines for Lung Cancer [13], surgery is the treatment modality with the highest curative potential for cases diagnosed at an early stage with localized disease. Given that 68% of early-stage NSCLC patients undergo surgery, a macrocosting approach was used to estimate the average annual hospitalization costs for lung cancer (ICD-10: C34) in 2023, based on procedures recommended by Brazilian therapeutic guidelines [6]: 04.12.05.010−2 – Wedge resection, tumor removal; 04.16.11.006−1 – Pulmonary segmentectomy in oncology; 04.16.11.001−0 – Pulmonary lobectomy in oncology. According to records from the Hospital Information System (SIH/DATASUS, 2023), a total of 2,769 hospitalizations met these criteria, with an average cost of $28,417.57 (95% CI: $20,667.45–$32,289.07).

Additionally, annual chemotherapy costs for Stage I and II lung cancer patients (ICD-10: C34) were also estimated. According to records from the Rio de Janeiro state database in the Ambulatory Information System (SIA/DATASUS, 2019): 224 out of 3.285 patients met the specified treatment criteria. The average annual chemotherapy cost was $5,429.87 (95% CI: $5,367.91–$5,491.89).

#### Advanced-stage disease (stage III or IV).

Unlike early-stage patients, most advanced-stage NSCLC patients are treated with chemotherapy, radiotherapy, or a combination of both [2]. For patients diagnosed at advanced stages, a microcosting study on lung cancer treatment was used. This study was conducted in a public oncology referral hospital in Brazil [32]. Based on a retrospective cohort of 277 patients, the study identified, quantified, and valued the direct healthcare costs of treatment, including medical consultations, diagnostic tests, medical procedures, and pharmaceutical expenses. After a mean survival of approximately 8.3 months, a Stage III or IV lung cancer patient had an annual treatment cost of approximately $69,392.59 (95% CI: $56,893.75–$81,894.80). Since these cost estimates were originally from 2014, they were adjusted in January 2025 based on the Brazilian healthcare inflation index (IPCA – Healthcare Services) for inclusion in the model.

#### Model assumptions

- In the model, false-negative cases were assumed to follow the same diagnostic pathway as in the no-screening scenario – that is, they would eventually be diagnosed through clinical suspicion. Therefore, their costs and outcomes were considered identical to the no-screening strategy, except for the additional cost of the AAC test.
- Treatment cost estimates are based only on the staging at diagnosis and do not account for disease progression over time.
- Due to limited available data on diagnostic accuracy, prevalence, utility, and survival values were collected and aggregated by lung cancer stage, categorized as: early-stage disease (Stage I and II), advanced-stage disease (Stage III and IV).
- Mortality for individuals without cancer at the time of screening follows age-related mortality trends in the general Brazilian population.

### Analytical model

To compare the respective costs and consequences of each strategy, with and without screening, and considering that this is a clinical screening scenario with implications for overall survival, a decision tree model coupled with a Markov model was constructed. The complete model accounted for the possible diagnostic pathways based on the accuracy (sensitivity and specificity) of the screening and the natural history of the disease, depending on whether the tumor is detected or not and at which stage. The model employed the Linked Evidence Approach (LEA), which evaluates the clinical effectiveness of a test based on systematically acquired evidence regarding its accuracy, its impact on clinical decision-making, and the effectiveness of the resulting treatment options [33].

**Fig 2:**
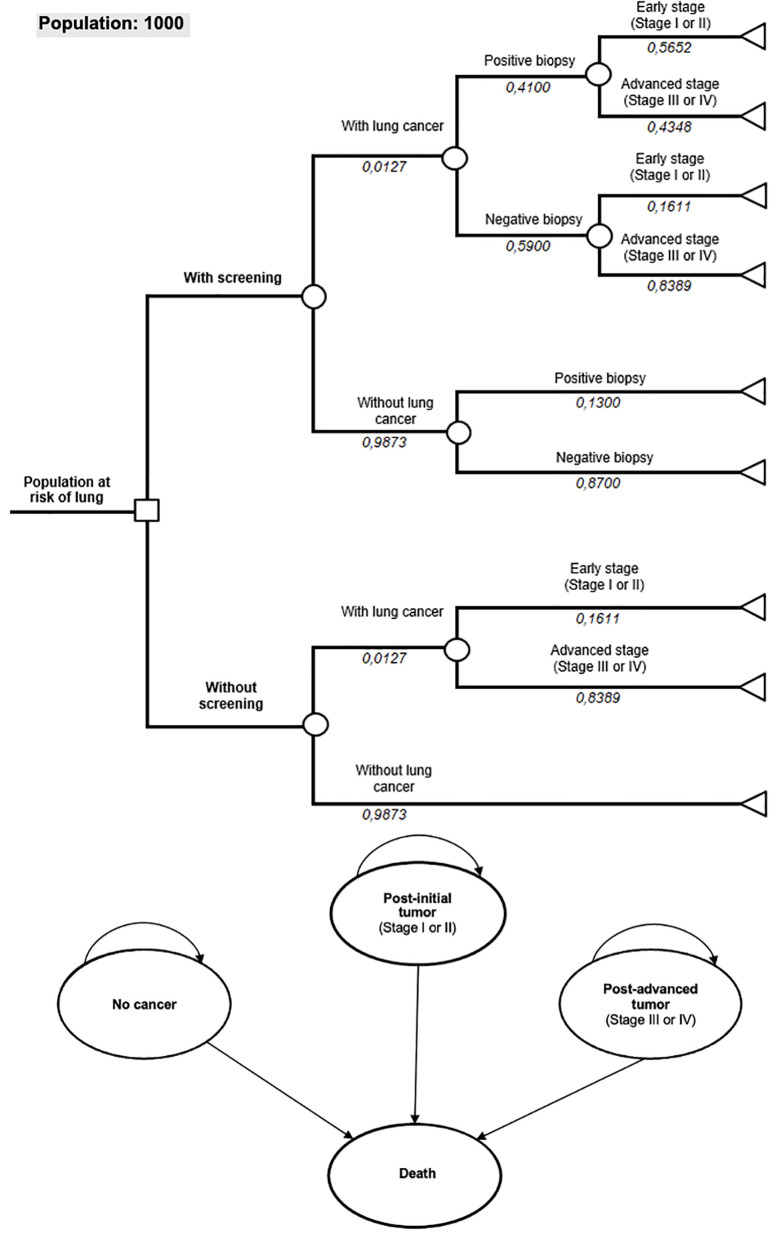
Representation of the decision tree structure of the model and the transition states of the Markov model.

From the screening strategy, the pathway follows according to the biopsy result until the diagnostic outcomes: early-stage lung cancer (Stage I and II), advanced-stage lung cancer (Stage III and IV), or absence of lung cancer (Fig 2). As illustrated, given the prevalence of lung cancer in the target population, the probability of the diagnostic pathway taken may be modified by the accuracy of the liquid biopsy (AAC test). To consider long-term outcomes, the decision tree was coupled with a Markov model. After defining the diagnostic events, which are the terminal nodes of the decision tree, each arm of the strategies under analysis feeds into a hypothetical cohort categorized by initial stage: no cancer, early-stage disease (Stage I or II), and advanced-stage disease (Stage III or IV). The Markov model was constructed with a one-year cycle length and applied the conventional half-cycle correction to account for transition probabilities within the model structure.

That staging state of the Markov model concerns the moment of diagnosis; that is, eventually any patient can progress to an advanced condition later and this is related with the survival probabilities adopted. Thus, despite the lack of specific cost data for each subsequent condition, our survival data according to staging at diagnosis considers this possibility of evolution.

In relation to diagnosis in advanced conditions (III or IV), this is already the current scenario in Brazil, where the majority of patients are unfortunately diagnosed in these stages with drastic reductions in survival prospects. We understand that biopsy would have minimal incremental gain when dealing with late diagnosis as well as clinical evaluation, not being the objective of our cost-effectiveness Transitions from early-stage to advanced-stage cancer were not explicitly modeled. This simplification was adopted due to the lack of robust epidemiological data on stage progression rates and reflects the assumption that most patients diagnosed at early stages either achieve remission or progress directly to recurrence or death without passing through an advanced-stage health state.

#### Transition probabilities.

Starting from the initial entry age into the model (mean of 61.9 years; 95% CI: 57.3 to 66.5 years), individuals without cancer transition to the death state according to the probabilities estimated from Brazilian general population mortality tables by age [34]. For individuals diagnosed with lung cancer, survival estimates available from cancer surveillance statistics were considered, where the five-year overall survival rate is reported as 53.5% for cases detected while the disease is still localized and 3.9% for patients with advanced-stage disease [2]. Based on these estimates, and assuming constant mortality rates, we derived the annual transition rates using the exponential method: Equation 1, where *r* is the annual mortality rate, *S*_*t*_ is the survival probability at time *t*, and t is the time in years (5 years in this model). These rates were then converted to annual transition probabilities using Equation 2, where *p* is the annual transition probability, *e* is the base of the natural logarithm, and *r* is the annual mortality rate from Equation 1, as described by Briggs. The model incorporated the exponential decline in overall survival according to the stage at which the tumor was detected, following this approach [35]. The five-year survival rates obtained from the curves considered in the model closely approximate those observed in the Brazilian context (S1 Fig).


$$r=\frac{-\ln\left(S_{t}\right)}{t}$$ (1)

$$p=1-e^{-r}$$ (2)

### Sensitivity analyses

Considering the point estimates, the range of values, their confidence intervals, and probability distributions, both deterministic sensitivity analyses (Tornado Analysis) and probabilistic sensitivity analyses (Monte Carlo simulations) were conducted. Due to the potential impact of overall survival estimates on the model results and their relationship with the local context, a scenario analysis was also performed using Brazilian data from a public oncology referral hospital (National Cancer Institute – INCA). INCA reported a five-year survival rate of 25.0% for diagnoses at early stages and 2.5% for advanced-stage cases [36]. According to the methods described, the point estimates and variations were adopted as presented in Table 1.

**Table 1:** Parameter data considered in the model.

Parameter description	Base case	Lower	Upper	Distribution	Source
Prevalence of lung cancer in the high-risk population	0.0127	0.0069	0.0231	Beta	Santos, 2016
Sensitivity of liquid biopsy (true positive)	0.4100	0.2900	0.5400	Beta	Jett, 2014
Specificity of liquid biopsy (true negative)	0.8700	0.8500	0.9000	Beta	Jett, 2014
Probability of initial stage (identified with screening)	0.5652	0.4225	0.6979	Beta	Jett, 2014
Probability of initial stage (not identified with screening)	0.1611	0.1452	0.1784	Beta	Jett, 2014
Annual mortality rate with diagnosis in the initial stage (Stage I-II)	0.1251	0.1001	0.1501	Uniform	Desantis, 2014
Annual mortality rate with diagnosis in the advanced stage (Stage III-IV)	0.6488	0.5195	0.7786	Uniform	Desantis, 2014
Utility without lung cancer	0.8580	0.8400	0.8760	Beta	Zimmermann, 2017
Utility with initial stage lung cancer	0.7720	0.7054	0.8386	Beta	Sturza, 2010
Utility with advanced lung cancer	0.5730	0.4417	0.7043	Beta	Sturza, 2010
Diagnostic cost	US$415.23	US$332.18	US$498.28	Uniform	Sigtap, 2019
Staging cost (Stage III-IV)	US$24.40	US$19.52	US$29.28	Uniform	Sigtap, 2019
Liquid biopsy cost	US$419.08	US$335.26	US$502.90	Uniform	Occleston, 2019
Treatment cost in the initial stage (Stage I-II)	US$874.65	US$608.37	US$993.81	Gamma	Sih-dataus, 2018
Treatment cost in the advanced stage (Stage III-IV)	US$2,135.81	US$1,751.11	US$2,520.61	Gamma	Kawat, 2017
Chemotherapy cost – initial stage (Stage I-II)	US$167.12	US$165.21	US$169.03	Gamma	Desantis, 2019
Table adjustment factor	2.84	1	3.12	Uniform	Brazil, 2016
Initial mean age	61.90	57.3	66.50	Normal	Santos, 2016
Annual discount rate	0.05	–	0.10	Uniform	Brazil, 2014

All analyses were conducted using Microsoft Excel®. The complete model with the data used is available in the public repository (github.com/XXXX) (S1 File). The CHEERS 2022 checklist was used to ensure the transparent and standardized reporting of the economic evaluation [37]. A completed version has been submitted as supplementary material (S2 Appendix).

## Results

### Incremental costs and outcomes

When considering the costs and consequences of implementing liquid biopsy for lung cancer screening in a high-risk population, a hypothetical cohort of 1,000 individuals resulted in an ICER of $ 75,435.63 per QALY gained. The analysis showed that the total cost for the no-screening strategy was $ 67,560, with 9.5899 QALYs, while the screening strategy had a total cost of $ 637,680, with 9.5975 QALYs. This resulted in an incremental cost of $ 570,120 and an incremental effectiveness of 7.56 QALYs.

Given the current Brazilian cost-effectiveness thresholds – $7,017.54 to $21,052.62 per QALY, as defined by the Brazilian Health Technology Assessment agency (CONITEC), the screening strategy with liquid biopsy was not considered cost-effective. These two thresholds reflect a range proposed by CONITEC, where the lower bound is applied to technologies with limited health gains or lower disease severity, and the upper bound is used in cases of higher severity or when substantial clinical benefits are expected. A univariate threshold analysis was conducted to explore at which prevalence the strategy could become cost-effective. The results indicated that the screening would only be cost-effective in contexts where lung cancer prevalence exceeds 4.0%. This prevalence lies outside the range used in the base-case analysis (as shown in Table 1) and was considered for exploratory purposes only.

### Sensitivity analyses

#### Deterministic.

**Fig 3:**
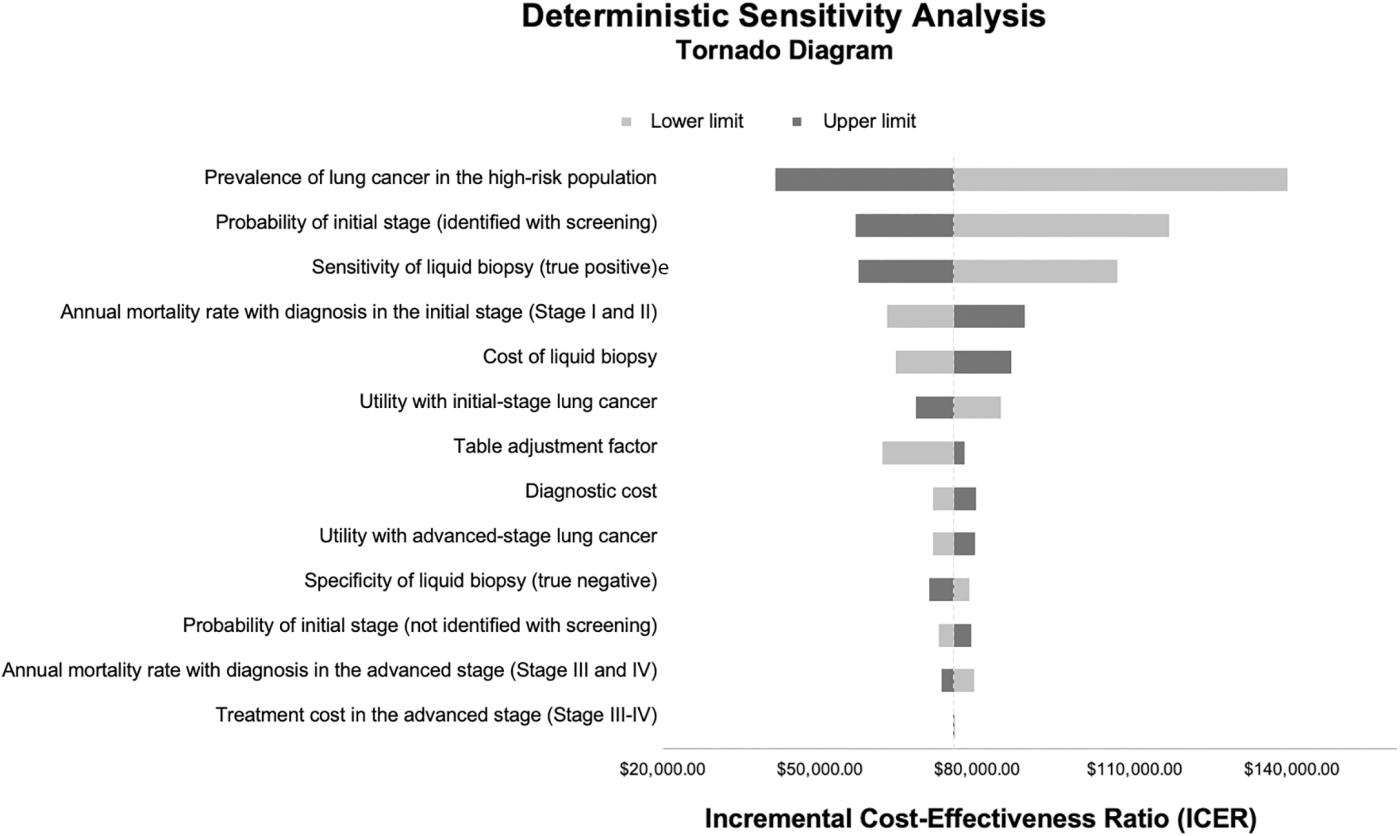
One-way sensitivity analysis with tornado plot. Ranges represent the impact of varying each input parameter individually on the ICER. For reference, the Brazilian willingness-to-pay threshold ranges from US$ 7,017.54 to US$ 21,052.62 per QALY.

As demonstrated in the Tornado Chart (Fig 3), the variables with the greatest impact on the results were lung cancer prevalence (ranging from 0.69% to 2.31%), the percentage of early-stage cases identified through screening (42.25% to 69.79%), the sensitivity of liquid biopsy (29% to 54%), the annual discount rate (0% to 10%), and mortality after diagnosis in early-stage cases (36.04% to 57.36% over five years). Considering the impact of these variations, the model proved to be robust, with an ICER range of $ 41,347.20 to $ 139,148.44 per QALY. Additionally, in an alternative scenario using five-year survival data from patient follow-up at a Brazilian public oncology referral hospital (National Cancer Institute – INCA) [38], the ICER increased to $ 144,968.82/QALY, with a range from $ 78,680.15 to $ 267,483.44, depending on lung cancer prevalence (2.31% to 0.69%, respectively).

Further details on the parameter-specific impact on the ICER, including full deterministic sensitivity analysis outputs, are available in the Excel model provided as supplementary material.

#### Probabilistic.

**Fig 4:**
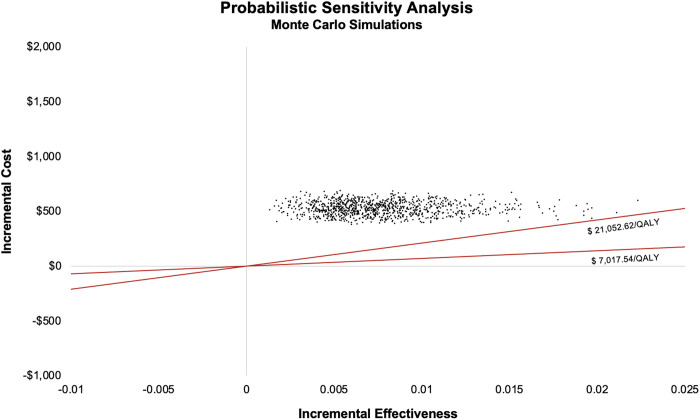
Scatterplot of probabilistic sensitivity analysis with 1000 Monte Carlo simulations.

With the 1,000 Monte Carlo simulations, assuming the data and distributions described in Table 1, a median ICER of $ 73,505.36/QALY was obtained ($ 33,843.92 to $ 204,439.90, using the 2.5th and 97.5th percentile method). In almost all simulated scenarios, the results are concentrated above the $ 7,017.54 and $ 21,052.62 thresholds, indicating a very low probability that the strategy would be cost-effective at either threshold (Fig 4).

**Fig 5:**
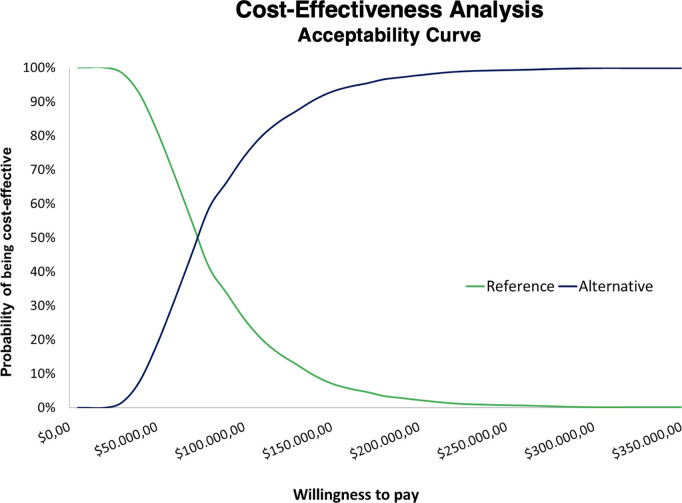
Cost-effectiveness acceptability curve (CEAC) based on 1,000 Monte Carlo simulations and varying willingness-to-pay thresholds.

In the cost-effectiveness acceptability curve (CEAC), a preference for the screening strategy emerges at willingness-to-pay thresholds above approximately $80,000 per QALY (Fig 5).

## Discussion

Based on the obtained estimates, the five-year relative survival rate of 18% for lung cancer could potentially increase to 56% with screening, allowing for early-stage disease detection. However, implementing liquid biopsy using an autoantibody test for lung cancer screening in high-risk populations also leads to increased costs. The cost-effectiveness analysis resulted in an ICER of $ 75,435.63 per quality-adjusted life year (QALY) gained. In the Brazilian context, where a cost-effectiveness threshold has not been officially defined, and considering a per capita GDP of approximately $ 5,263.15, the technology would only be considered cost-effective if lung cancer prevalence exceeded 15% which is five times higher than the current prevalence in Brazil (below 3%) [24]. The five-year survival rate, with only 53% for early-stage cases and 4% for advanced-stage cases, combined with the low sensitivity of the technology, reduces the benefits of early diagnosis, despite its clinical significance. The autoantibody test evaluated in this study has a sensitivity of 41% and a specificity of approximately 90%, according to the manufacturer.

A cost-effectiveness study by Edelsberg et al. [21], with differences in study population and comparator, evaluated the autoantibody test as an aid in the early diagnosis of lung cancer in patients with incidentally detected pulmonary nodules (8–30 mm) at intermediate lung cancer risk under CT surveillance. In their study, individuals with a positive autoantibody test were referred for tissue biopsy to confirm malignancy, while those with negative results remained under CT surveillance at 4, 10, and 21 months. In the comparator arm, patients with negative CT results followed the same surveillance protocol. The authors concluded that the autoantibody test was cost-effective, reporting a sensitivity of 28% and specificity of 98%, with an ICER of $ 18,454/life-year gained and $ 24,833/QALY gained.

The present study has several methodological strengths. Diagnostic accuracy parameters were derived from a systematic review encompassing the principal medical literature databases, with efforts to minimize selection bias through a paired and independent review process. Cost and effectiveness estimates were tailored to the Brazilian context, prioritizing the use of national data sources and official statistics whenever available.

The analytical model captured both short- and long-term impacts of the screening strategy: a decision tree was applied for initial outcomes, followed by a Markov model to simulate disease progression over time. Survival data were extrapolated using parametric functions calibrated to closely reflect observed outcomes in the Brazilian population [39].

In addition to a comprehensive search for point estimates, deterministic and probabilistic sensitivity analyses were conducted to evaluate parameter uncertainty. The most influential variables in the model were the time horizon, the incidence rate of lung cancer, and the proportion of early-stage diagnoses. Shorter time horizons yielded higher ICERs, as they failed to capture long-term health gains. Conversely, longer time horizons resulted in lower ICERs by incorporating a greater number of QALYs gained from early detection and treatment. These findings suggest that improvements in operational parameters or cost reductions of the screening technology would have limited influence on the ICER.

Across all simulated scenarios, the strategy did not demonstrate cost-effectiveness. However, changes in the availability, effectiveness, and cost of treatments, especially for early-stage lung cancer, may substantially affect the cost-effectiveness of the screening approach. Technological or therapeutic advances that enhance survival or reduce treatment costs have the potential to increase health benefits associated with early detection and reduce the ICER.

However, this study also presents several limitations that should be acknowledged. First, the model does not account for indolent cases of lung cancer, those that, if incidentally detected, would progress so slowly that they would never reach clinical significance. These overdiagnosed cases may lead to intangible physical and emotional harm, as such tumors typically have a highly favorable prognosis and would not result in mortality.

Second, the Brazilian diagnostic and therapeutic guidelines for lung cancer do not fully define the characteristics of the at-risk population. As a result, it was necessary to use information from unofficial national sources and international literature. Nevertheless, data from the Brazilian Surveillance System for Risk and Protective Factors for Chronic Diseases through Telephone Survey (VIGITEL) were used to estimate the number of smokers by age group. The proportions observed in the 45–54 (11.1%) and 55–64 (12.3%) age groups were consistent with the at-risk populations described in the literature. Due to the absence of robust national estimates, certain assumptions were adopted in the construction of the model and are transparently described in the Methods section.

Third, adverse events related to diagnostic procedures were not incorporated into the model. However, it is important to recognize that the multiple procedures involved in establishing a lung cancer diagnosis may affect patients’ quality of life and utility values.

Fourth, the analysis did not include low-dose computed tomography (LDCT) as a comparator, although this technology represents an emerging and potentially valuable diagnostic strategy. Instead, the analysis focused on clinical diagnosis, as it reflects the most commonly used approach in the Brazilian healthcare system, thereby enhancing the relevance and applicability of the findings.

Fifth, the model focused exclusively on non-small cell lung cancer (NSCLC), excluding small cell lung cancer (SCLC), which, although less prevalent, may also be detected through screening. This simplification was based on the higher prevalence of NSCLC and the validation scope of the EarlyCDT-Lung® test but is recognized as a limitation.

Lastly, updated cost data for the autoantibody test used as a form of liquid biopsy were not available. According to the manufacturer’s website, pricing varies depending on the region where the test is administered. Therefore, the analysis adopted the previously reported manufacturer’s suggested price in U.S. dollars, converted into Brazilian reais, as referenced in earlier publications.

## Conclusion

Based on the results of the economic model, it is observed that although the autoantibody test as a liquid biopsy is a useful tool for the early detection of lung cancer, its adoption as a screening strategy does not present itself as a cost-effective alternative for high-risk populations in Brazil. Without substantial changes in the sensitivity values and cost of the liquid biopsy test analyzed here, screening would only become cost-effective in scenarios where lung cancer prevalence exceeds 4% (based on threshold analysis), a situation that is unlikely in the Brazilian screening context, where prevalence remains below 3%.

## Supporting information

S1 Fig Survival curves according to the diagnosis stage. (TIF)

S1 Appendix Preferred Reporting Items for Systematic Reviews and Meta-Analyses (PRISMA) checklist. (DOCX)

S1 File Excel-based decision-analytic model used for the cost-effectiveness analysis of liquid biopsy for lung cancer screening in Brazil. (XLSM)

S2 Appendix Consolidated Health Economic Evaluation Reporting Standards (CHEERS) 2022 checklist. (DOCX)
