# Supplementary figures and images for: Cost-effectiveness assessment of liquid biopsy for early detection of lung cancer in Brazil
Source: PLoS One. 2025 Jul 29;20(7):e0328631. doi: 10.1371/journal.pone.0328631 (PMC12306772; doi:10.1371/journal.pone.0328631)

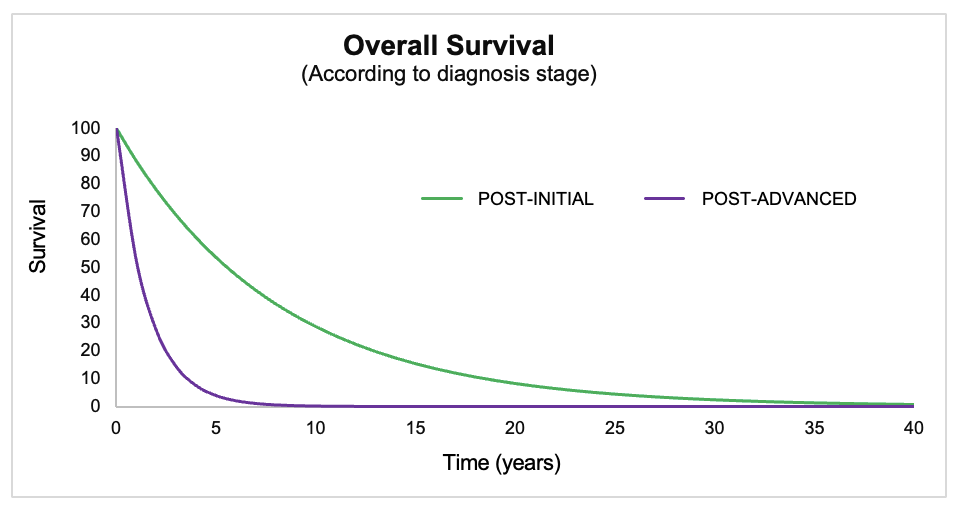

Supplement: S1 Fig — (TIF) [file pone.0328631.s001.tif]
