# Supplementary material for: Cost-effectiveness assessment of liquid biopsy for early detection of lung cancer in Brazil
Source: PLoS One. 2025 Jul 29;20(7):e0328631. doi: 10.1371/journal.pone.0328631 (PMC12306772; doi:10.1371/journal.pone.0328631)
Supplement: S2 Appendix — (DOCX) [file pone.0328631.s004.docx]

**Consolidated Health Economic Evaluation Reporting Standards (CHEERS) 2022 Checklist**

**Title**

**1. Title**

Identify the study as an economic evaluation and specify the interventions being compared.

Answer: This study, titled “Cost-effectiveness assessment of liquid biopsy for early detection of lung cancer in Brazil,” is a model-based economic evaluation comparing the use of liquid biopsy (EarlyCDT-Lung® test) as a screening strategy for early detection of lung cancer in a high-risk population versus no screening.

**Abstract**

**2. Abstract**

Provide a structured summary that highlights context, key methods, results, and alternative analyses.

Answer: The abstract summarizes the background and objective of evaluating the cost-effectiveness of liquid biopsy (EarlyCDT-Lung® test) as a screening strategy for early lung cancer detection in Brazil. A decision tree and Markov model were used to simulate short- and long-term outcomes in a hypothetical cohort of 1,000 high-risk individuals. Effectiveness was measured in QALYs, and costs were estimated from the perspective of the Brazilian public health system (SUS). The ICER for the screening strategy was $75,435.63 per QALY gained, well above the Brazilian willingness-to-pay thresholds ($7,017.54 to $21,052.62). Sensitivity analyses demonstrated that the model was robust, and the screening strategy would only be cost-effective if lung cancer prevalence exceeded 4.0%. The study concludes that, under current conditions, liquid biopsy is not a cost-effective strategy in the Brazilian context.

**Introduction**

**3. Introduction: Background and Objectives**

Give the context for the study, the study question, and its practical relevance for decision making in policy or practice.

Answer: Lung cancer is the leading cause of cancer mortality in Brazil, often diagnosed at advanced stages due to lack of structured screening programs in the public health system. While low-dose computed tomography (LDCT) is recommended internationally for early detection, its adoption in Brazil is limited due to logistical, financial, and operational barriers. Liquid biopsy using autoantibody detection (EarlyCDT-Lung®) offers a less invasive, more accessible screening alternative. This study evaluates whether implementing liquid biopsy as a screening strategy in high-risk individuals is cost-effective under the Brazilian public healthcare system (SUS), using economic modeling to inform decision-makers and guide resource allocation.

**Methods**

**4. Health economic analysis plan**

Indicate whether a health economic analysis plan was developed and where available.

Answer: A formal health economic analysis plan was not published separately. However, all modeling assumptions and methodological decisions—including model structure, comparators, data sources, perspective, time horizon, and types of sensitivity analyses—were pre-specified and transparently reported in the Methods section of the manuscript, following current best practices for economic evaluations.

**5. Study population**

Describe characteristics of the study population (such as age range, demographics, socioeconomic, or clinical characteristics).

Answer: The target population for the model was defined as all individuals at high risk for lung cancer, meeting the following criteria, established in cohort studies and validated in national and international guidelines: Aged over 55 years; Absence of significant respiratory symptoms; Smoking history of more than 30 pack-years (current smokers or those who quit within the last 15 years). For this model, NSCLC was used as the reference, given its representativeness of approximately 90% among different lung cancer types.

**6. Setting and location**

Provide relevant contextual information that may influence findings.

Answer: The study was conducted from the perspective of the Brazilian public healthcare system (Sistema Único de Saúde – SUS), considering the availability, cost structure, and diagnostic capabilities in Brazil. The analysis used national cost databases, official tariffs, and epidemiological data relevant to the Brazilian context.

**7. Comparators**

Describe the interventions or strategies being compared and why chosen.

Answer: The model compared two strategies: (1) No screening, where lung cancer is diagnosed based on clinical suspicion. In this scenario, patients with suspected early-stage disease (Stage I or II) are assumed to undergo low-dose computed tomography (LDCT) and PET-CT, while patients with suspected advanced disease (Stage III or IV) may undergo bronchoscopy and percutaneous biopsy for diagnosis and staging. (2) Screening with liquid biopsy using the EarlyCDT-Lung® test, in which individuals with a positive test result are referred for confirmatory and staging procedures. Although specific diagnostic tests were not explicitly modeled, these typically include LDCT, PET-CT, bronchoscopy, and biopsy, according to national guidelines. The comparator reflects current clinical practice in the Brazilian public healthcare system, which lacks a structured screening program.

**8. Perspective**

State the perspective(s) adopted by the study and why chosen.

Answer: The analysis adopted the perspective of the Brazilian public healthcare system (SUS), considering only direct medical costs such as screening, diagnosis, treatment, and follow-up. Non-medical and indirect costs were not included.

**9. Time horizon**

State the time horizon for the study and why appropriate.

Answer: The model adopts a lifetime horizon to capture the full costs and health outcomes associated with lung cancer detection and treatment. Brazilian life tables from 2017 were used to simulate disease progression based on the average age of the screening cohort. This approach reflects the natural history of lung cancer, which has a significant impact on overall survival and quality of life, justifying the need for a long-term analytical perspective.

**10. Discount rate**

Report the discount rate(s) and reason chosen.

Answer: Following Brazilian methodological guidelines for economic evaluations, an annual discount rate of 5% was applied to both costs and outcomes in the base-case analysis. To assess the impact of discounting assumptions, a range from 0% to 10% was explored in the deterministic sensitivity analysis.

**11. Selection of outcomes**

Describe what outcomes were used as the measure(s) of benefit(s) and harm(s).

Answer: The primary health outcome was quality-adjusted life years (QALYs), which combines both survival and quality of life. This measure allows comparisons across health interventions and is consistent with Brazilian and international guidelines for economic evaluations.

**12. Measurement of outcomes**

Describe how outcomes used to capture benefit(s) and harm(s) were measured.

Answer: Outcomes were measured through a Markov model that incorporated different health states (no cancer, early-stage lung cancer, advanced-stage lung cancer, and death). Transition probabilities and survival estimates were derived from epidemiological data and literature, with QALY weights applied according to disease stage.

**13. Valuation of outcomes**

Describe the population and methods used to measure and value outcomes.

Answer: Utility values used to estimate QALYs were obtained from published studies reporting quality-of-life weights for early- and advanced-stage lung cancer. These values reflect international literature and were applied to the Brazilian context due to the lack of local utility data.

**14. Measurement and valuation of resources and costs**

Describe how costs were valued.

Answer: Costs were calculated from the SUS perspective using official government sources (SIGTAP, SIA/SUS, SIH/SUS) and published Brazilian cost studies. Unit costs for diagnostic tests, treatments, and procedures were applied in 2024 BRL and converted to USD. The model included direct medical costs only.

**15. Currency, price date, and conversion**

Report the dates of the estimated resource quantities and unit costs, plus the currency and year of conversion.

Answer: Costs were collected in Brazilian currency (R$) at 2024 values and converted to US dollars ($) at the exchange rate of R$5.70 per USD on February 14, 2025. Official sources were used to obtain SUS reimbursement values. The identification and quantification of cost items followed the NLST protocol (USA, 2002–2010) and the Brazilian Diagnostic and Therapeutic Guidelines for Lung Cancer. Although treatment effectiveness was not the study focus, costs related to treatment procedures were considered based on cancer staging.

**16. Rationale and description of model**

If modeling is used, describe in detail and why used. Report if the model is publicly available and where it can be accessed.

Answer: A combined decision tree and Markov model was used to simulate both short- and long-term health and cost outcomes associated with the use of liquid biopsy versus no screening. The decision tree captured initial diagnostic outcomes, while the Markov model simulated long-term disease progression, survival, and QALY accumulation across health states. This modeling structure is appropriate for chronic diseases with long-term consequences, such as lung cancer.

**17. Analytics and assumptions**

Describe any methods for analyzing or statistically transforming data, any extrapolation methods, and approaches for validating any model used.

Answer: Transition probabilities were derived from survival data using the exponential distribution, assuming constant hazard rates. Utilities and costs were assigned by health state and discounted annually. Assumptions included full adherence to diagnostic follow-up after a positive test and the absence of structured screening in the comparator arm. A half-cycle correction was applied in the Markov model.

**18. Characterizing heterogeneity**

Describe any methods used for estimating how the results of the study vary for subgroups.

Answer: No subgroup analyses were conducted.

**19. Characterizing distributional effects**

Describe how impacts are distributed across different individuals or adjustments made to reflect priority populations.

Answer: Deterministic (univariate) sensitivity analyses were performed to identify influential parameters. A probabilistic sensitivity analysis (PSA) with 1,000 Monte Carlo simulations was also conducted, incorporating uncertainty around all input parameters using appropriate probability distributions (beta, gamma, and log-normal).

**20. Characterizing uncertainty**

Describe methods to characterize any sources of uncertainty in the analysis.

Answer: Internal validity was ensured by checking model logic and consistency of outcomes. Transition probabilities and survival outputs were calibrated against published epidemiological data and Brazilian life tables. The model structure and assumptions were based on best practices and consistent with similar published models in the literature.

**21. Approach to engagement with patients and others affected by the study**

Describe any approaches to engage patients or service recipients, the general public, communities, or stakeholders (eg, clinicians or payers) in the design of the study.

Answer: This study did not involve direct engagement with patients, the public, or other stakeholders in the design, conduct, or reporting of the analysis.

**Results**

**22. Study parameters**

Report all analytic inputs (eg, values, ranges, references) including uncertainty or distributional assumptions.

Answer: All model parameters are detailed in Table 1 of the manuscript, with base-case values, lower and upper bounds, probability distributions, and data sources. Key parameters include: Prevalence of lung cancer in the high-risk population 1.27% (range: 0.69%–2.31%, Beta distribution; source: Santos, 2016); Sensitivity and specificity of liquid biopsy 41.0% (29.0%–54.0%) and 87.0% (85.0%–90.0%) respectively (Beta; Jett, 2014); Probability of early-stage diagnosis with screening 56.5% (42.3%–69.8%, Beta; Jett, 2014). Utility values: 0.8580 without cancer, 0.7720 with early-stage lung cancer, 0.5730 with advanced cancer (Beta; Zimmermann, 2017; Sturza, 2010). Mortality rates: 12.5% annually for early-stage and 64.8% for advanced-stage diagnosis (Beta; Desantis, 2014). Costs: Diagnostic US$415.23 (Uniform; Sigtap, 2019), Staging (Stage III-IV) US$24.40 (Uniform; Sigtap, 2019), Liquid biopsy US$419.08 (Uniform; Oclelston, 2019). Treatment: Early stage US$874.65 (range: 608.37–993.81, Gamma; Sih-datasus, 2018), Advanced stage US$2,135.81 (range: 1,751.11–2,520.61, Gamma; Kawat, 2017), Chemotherapy for early stage: US$167.12 (Gamma; Desantis, 2019). Discount rate: 5% per year (range: 0%–10%, Uniform; Brazil, 2014). Initial age: 61.9 years (95% CI: 57.3–66.5; Normal; Santos, 2016). All parameters used in the probabilistic sensitivity analysis were assigned appropriate probability distributions: Beta for probabilities and utilities, Gamma for skewed cost data, and Uniform or Normal where applicable.

**23. Summary of main results**

Report the mean values for the main categories of costs and outcomes of interest and summarize them in the most appropriate overall measure.

Answer: Uncertainty was assessed using both deterministic and probabilistic sensitivity analyses. In the deterministic analysis, variations in single parameters within predefined ranges (Table 1) showed that the model was most sensitive to lung cancer prevalence, test sensitivity, and the proportion of early-stage diagnoses. These variations resulted in ICERs ranging from $41,347.20 to $139,148.44 per QALY. An additional scenario using real-world survival data from a Brazilian public cancer center (INCA) increased the ICER to $144,968.82, with a broader range depending on prevalence ($78,680.15 to $267,483.44). The probabilistic sensitivity analysis (PSA) was conducted with 1,000 Monte Carlo simulations, applying appropriate probability distributions for each parameter. The cost-effectiveness acceptability curve (CEAC) showed that the screening strategy had a near-zero probability of being cost-effective under Brazilian willingness-to-pay thresholds.

**24. Effect of uncertainty**

Describe how uncertainty about analytic judgments, inputs, or projections affects findings. Report the effect of choice of discount rate and time horizon, if applicable.

Answer: No direct engagement with patients or stakeholders was conducted during the study; therefore, no impact from such involvement on the study results is applicable.

**25. Effect of engagement with patients and others affected by the study**

Report on any difference patient/service recipient, general public, community, or stakeholder involvement made to the approach or findings of the study.

Answer: An alternative modeling scenario was explored by modifying the source of survival data. In this scenario, five-year survival rates from a Brazilian public oncology center (INCA) were used in place of international estimates. This structural change in transition probabilities significantly affected results, increasing the ICER to $144,968.82, with a range from $78,680.15 to $267,483.44, depending on cancer prevalence. No other changes to model structure were tested.

**Discussion**

**26. Study findings, limitations, generalizability, and current knowledge**

Report key findings, limitations, ethical, or equity considerations not captured and how these could impact patients, policy, or practice.

Answer: This study found that implementing liquid biopsy (EarlyCDT-Lung®) as a screening strategy for early detection of lung cancer in a high-risk Brazilian population results in an incremental cost-effectiveness ratio (ICER) of $75,435.63 per QALY, which is well above the Brazilian cost-effectiveness thresholds of $7,017.54 to $21,052.62 per QALY. Across all scenarios tested, including sensitivity and threshold analyses, the screening strategy was not cost-effective under current conditions. Only at a prevalence above 4%, which exceeds the range reported in national data, would the strategy become potentially cost-effective. The model demonstrated robustness in deterministic and probabilistic sensitivity analyses. The main drivers of uncertainty were lung cancer prevalence, test sensitivity, and the proportion of early-stage cases detected. A scenario using local survival data (from INCA) showed a considerable increase in the ICER, reinforcing the model’s sensitivity to epidemiological context. Limitations include the absence of local utility data, the assumption of full adherence to diagnostic follow-up after a positive test, and the exclusion of indirect or non-medical costs. Additionally, specific confirmatory tests after liquid biopsy were not modeled individually but were assumed to follow national clinical guidelines. Despite these limitations, the findings are relevant to Brazilian policy decision-makers and may inform discussions about incorporating new diagnostic technologies into public health strategies. Generalizability to other countries may be limited due to differences in disease prevalence, health system structure, and cost data.

**Other Relevant Information**

**27. Source of funding**

Describe how the study was funded and any role of the funder in the identification, design, conduct, and reporting of the analysis.

Answer: This study was supported by the Brazilian Ministry of Health through the PROADI-SUS program (Programa de Apoio ao Desenvolvimento Institucional do SUS). The funder had no role in the design, conduct, analysis, or interpretation of the study results.

**28. Conflicts of interest**

Report authors’ conflicts of interest according to journal or International Committee of Medical Journal Editors requirements.

Answer: The authors declare no conflicts of interest related to this study.
